# Comparison of gluteal muscle central activation in individuals with and without patellofemoral pain

**DOI:** 10.3389/fphys.2025.1535141

**Published:** 2025-02-18

**Authors:** Kai-Yu Ho, Michael Carpio, John Donohue, Jacob Kissman, Jing Nong Liang

**Affiliations:** Department of Physical Therapy, University of Nevada, Las Vegas, Las Vegas, NV, United States

**Keywords:** patellofemoral pain, central activation, gluteal muscles, frontal plane kinematics, superimposed burst technique

## Abstract

Patellofemoral pain (PFP) is often linked to knee valgus during weight-bearing activities, commonly attributed to gluteal muscle weakness. However, recent research suggests that central nervous system adaptations may also influence muscle function and movement patterns in individuals with PFP. This study compared the central activation ratio (CAR) of the gluteus medius and gluteus maximus between individuals with and without PFP, and assessed the associations between gluteal CAR, frontal plane projection angle of the trunk and lower extremity, and knee function. Twelve individuals without PFP and 10 individuals with PFP participated. We tested CAR of the gluteal muscles with a superimposed burst protocol during a maximum voluntary isometric contraction and evaluated frontal plane kinematics of the trunk and lower extremities during five single leg tasks. Participants with PFP also completed the Anterior Knee Pain Scale (AKPS). Independent t-tests compared CAR between groups, and Pearson correlation coefficients evaluated the associations between CAR, frontal plane kinematics, and AKPS. Individuals with PFP tended to have lower gluteus maximus CAR, though the difference was not statistically significant (PFP: 90.8% ± 7.0%, Control: 94.4% ± 3.0%; p = 0.067). CAR of both the gluteus maximus (R = 0.790, p = 0.003) and gluteus medius (R = 0.584, p = 0.038) were significantly correlated with AKPS scores, and gluteus maximus CAR was associated with trunk lean angle during single leg landing (R = 0.533, p = 0.006). Our data suggest that higher gluteal CAR is associated with better function in individuals with PFP. Lower gluteus maximus CAR contributes to ipsilateral trunk lean during single leg landing, potentially to reduce external hip moments and muscle demand.

## 1 Introduction

Research on patellofemoral pain (PFP) has highlighted its association with dynamic knee valgus, a movement impairment characterized by a combination of femoral internal rotation and adduction, knee abduction, tibial external rotation, and ankle pronation during weight-bearing activities (Wilczyński et al., 2020). Such altered movement patterns can result in lateral tracking of the patella and increased loading on the lateral patella, which is thought to contribute to PFP (Powers et al., 2017; Willy et al., 2019; Wilczyński et al., 2020). Given that weakness in the hip muscles is believed to contribute to dynamic knee valgus, physical therapy interventions for PFP often emphasize strengthening the hip abductors and external rotators (Powers et al., 2017; Willy et al., 2019). However, while hip muscle strengthening programs are commonly prescribed, and may successfully reduce pain and hip muscle weakness in the short term, evidence suggests that these interventions may lack efficacy in maintaining long-term improvements and may not adequately correct faulty movement patterns during weight-bearing activities (Willy et al., 2019). This suggests that factors beyond hip muscle strength alone may contribute to the persistence of movement deficits in individuals with PFP.

Central nervous system adaptations in individuals with PFP have been shown in more recent literature, including cortical reorganization of the primary motor cortex, altered spinal reflex excitability, and inhibition of the quadriceps muscles (On et al., 2004; Rio et al., 2016; de Oliveira Silva et al., 2017; Te et al., 2017; Pazzinatto et al., 2019; Liang et al., 2021; Ho et al., 2022; Waiteman et al., 2022). The altered neural pathways observed in persons with PFP may impair their ability to voluntarily activate the affected muscles, which can be crucial for generating the necessary forces to maintain proper joint mechanics and movement patterns during weight-bearing tasks. However, current interventions primarily focus on strengthening weakened muscles rather than directly addressing the underlying altered neural pathways (Bolgla et al., 2016; Willy et al., 2019). This approach might explain the limited long-term effectiveness of conventional rehabilitation for PFP. The impairment in voluntary activation of the gluteal muscles may help explain why hip strengthening protocols alone do not consistently improve muscle strength or correct dynamic knee valgus during weight-bearing activities (Bolgla et al., 2016; Willy et al., 2019). For instance, gluteal muscle inhibition, characterized by the nervous system’s inability to fully activate gluteal muscles, may explain the limited success of strengthening interventions (Glaviano et al., 2019; Glaviano and Norte, 2022; Ho et al., 2022). Addressing the underlying neural adaptations, in addition to muscular strengthening, may be required to effectively restore proper joint mechanics and alleviate symptoms.

Voluntary muscle activation can be experimentally quantified using the central activation ratio (CAR) through the superimposed burst (SIB) technique (Glaviano et al., 2019; Glaviano and Norte, 2022). SIB involves applying an exogenous electrical stimulus percutaneously following a maximum voluntary isometric contraction (MVIC). This method allows for the determination of CAR, which represents the ratio of volitionally activated motor units to the total available motor units within a specific muscle (Glaviano et al., 2019; Glaviano and Norte, 2022). By utilizing the SIB technique during MVIC, researchers can assess the extent of muscle activation and identify potential deficits in neuromuscular function.

Recent findings suggest that diminished central activation of the gluteus medius in females with PFP is associated with increased hip adduction during single leg squat and fear-avoidance beliefs (Glaviano and Norte, 2022). However, this study did not include a control group without PFP (Glaviano and Norte, 2022). In addition, an unpublished study (Samuel, 2022) reported that females with PFP had a reduced CAR of the gluteus medius compared to pain-free controls, though no associations were found with knee valgus angle during forward step-down. Notably, the unpublished reports by Samuel focused solely on the gluteus medius, leaving the gluteus maximus unexamined, and assessed CAR in relation to knee valgus during only one weight-bearing task. Females with PFP frequently exhibit weakness in both the gluteus medius and gluteus maximus muscles compared to pain-free individuals, as demonstrated by isometric and isokinetic strength assessments (Van Cant et al., 2014). This finding suggests that hip muscle weakness is not limited to a specific type of muscle contraction or a single gluteal muscle group. Individuals with PFP also demonstrate deficits across multiple aspects of muscle performance, including hip abductor rate of force development, hip muscle power, and dynamic hip strength, such as the force generated during repetition maximum tests (Nunes et al., 2018; Nunes et al., 2019). A wide range of gluteal muscle CAR in females with PFP have also been reported (Glaviano and Norte, 2022), potentially suggesting variability in neuromuscular responses to injury or pain, with a subset experiencing gluteal muscle inhibition. The current literature lacks comprehensive research comparing central activation of both gluteus maximus and gluteus medius muscles between individuals with and without PFP, and exploring the associations between gluteal muscle activation, frontal plane kinematics of the trunk and lower limbs during various weight-bearing tasks, and related functional outcomes. This knowledge gap highlights the need for further studies to better understand the neural mechanisms driving PFP and their impact on movement patterns and functional performance.

Therefore, the primary aim of this study was to compare the CAR of the gluteus medius and gluteus maximus between individuals with and without PFP. A secondary aim was to examine the relationships between CAR of the gluteal muscles and the frontal plane projection angle (FPPA) of the trunk and lower extremities during five weight-bearing activities and patellofemoral joint function. We hypothesized that individuals with PFP would exhibit a lower CAR in both the gluteus medius and gluteus maximus compared to those without PFP. Additionally, we hypothesized that lower gluteal CAR would correlate with altered FPPA of the trunk and lower extremities and reduced function.

## 2 Materials and methods

### 2.1 Participants

A sample size calculation was performed prior to the start of the study, indicating that 9 participants per group (PFP and non-PFP) were required to achieve 80% statistical power with a Type I error of 0.05. This estimation was based on prior research examining gluteus medius CAR and sought to detect a potential 8% difference between the two groups (Glaviano and Norte, 2022; Samuel, 2022). Therefore, we aimed to recruit at least 9 participants with PFP and 9 pain-free controls.

The inclusion criteria for the PFP group involved individuals aged 18–45 years who experienced PFP, specifically peri- or retro-patellar pain lasting at least 3 months. A physical examination was conducted to ensure that participants′ pain was not caused by other sources. This examination included palpation around the patellofemoral joint and a patellar compression test, where the patella was pressed while the knee was extended. If the pain did not originate from the patellofemoral joint, participants were excluded from the study (Ho et al., 2021; Ho et al., 2024). For the control group, participants aged 18–45 years who did not have PFP were included. Exclusion criteria for both PFP and control groups included those with a history of traumatic patellar dislocation, previous knee surgeries, or pregnancy.

The Institutional Review Board at the University of Nevada, Las Vegas approved this study (protocol# UNLV-2023-86). Recruitment occurred between 2023 and 2024 via local physical therapy clinics, the university, and other local organizations around Las Vegas. All participants who met the inclusion criteria and consented to the study received detailed information regarding the procedures, risks, and benefits before participating.

### 2.2 Procedures

All participants completed two examination sessions on the same day: the first assessed frontal plane kinematics during weight-bearing activities, followed by an evaluation of central activation of the gluteal muscles. In participants with PFP, the assessment was performed on the symptomatic or more symptomatic limb. For those in the control group, the dominant leg was studied, which was identified as the preferred limb used for kicking a ball (van Melick et al., 2017).

#### 2.2.1 Pain and function assessment

Before assessing frontal plane kinematics during weight-bearing activities, participants with PFP were asked to report their pain and functional status using validated self-report measures. The Numeric Pain Rating Scale (NPRS) was employed to evaluate their usual pain during daily activities, with 0 representing no pain and 10 representing extreme pain. NPRS has been shown to be valid, reliable and appropriate for use in clinical practice (Williamson and Hoggart, 2005). To assess patellofemoral joint function, the Anterior Knee Pain Scale (AKPS) was administered in participants with PFP. The AKPS is a reliable self-report tool designed specifically for evaluating function in individuals with PFP. It includes 13 weighted questions, with a total score of 100 indicating no disability. Higher scores reflect better functional status (Crossley et al., 2002). All participants were also asked to report their physical activity levels using the Global Physical Activity Questionnaire (GPAQ), developed by the World Health Organization to assess physical activity and sedentary behaviors. The GPAQ consists of 16 questions covering three domains: work-related activity, travel, and recreational activities. It has been validated as an effective tool for measuring moderate to vigorous physical activity and shows a strong correlation with the International Physical Activity Questionnaire (Bull et al., 2019).

#### 2.2.2 Assessment of frontal plane kinematics during weight-bearing activities

To capture frontal plane kinematics of the trunk and lower extremities for each participant, spherical matte stickers were used as markers. These markers were placed on the manubrium of the sternum, bilateral anterior superior iliac spines (ASIS), bilateral patellae, and bilateral mid-talus. Participants were then videotaped performing a series of weight-bearing tasks: single leg squat, single leg hop, single leg landing, forward step-down, and lateral step-down. All movements were recorded using an iPhone 13 Pro Max camera (Apple Inc., Cupertino, CA, United States) set to 30 frames per second in high definition with a 1x zoom. For all participants, the camera was mounted on a tripod positioned 0.71 m above the ground and 3.1 m in front of the participants to ensure consistent two-dimensional (2D) frontal plane measurements.

For the single leg squat test, participants stood on their testing limb, performed a squat, flexing their affect knee until they reached their maximal knee flexion without loss of balance. During the single leg hop test, they were instructed to hop as far as possible on the testing leg (Ho et al., 2019). In the single leg landing task, participants started on their testing leg atop a 6 inch platform, stepped forward, and landed on the same leg. The forward step-down test required participants to stand on their testing leg on a 30 cm box, lower the other leg forward to touch the ground, and then return to the starting position within a 2-second period (Lee and Powers, 2014). For the lateral step-down test, participants stood on their testing leg on a 30 cm box, lowered the other leg to the side to touch the ground, and returned to the starting position within 2 seconds (Ho et al., 2024). Each task was performed three times, with 3-minute rest intervals between tasks. Additional breaks were allowed upon request.

#### 2.2.3 Assessment of central activation of the gluteal muscles

**FIGURE 1:**
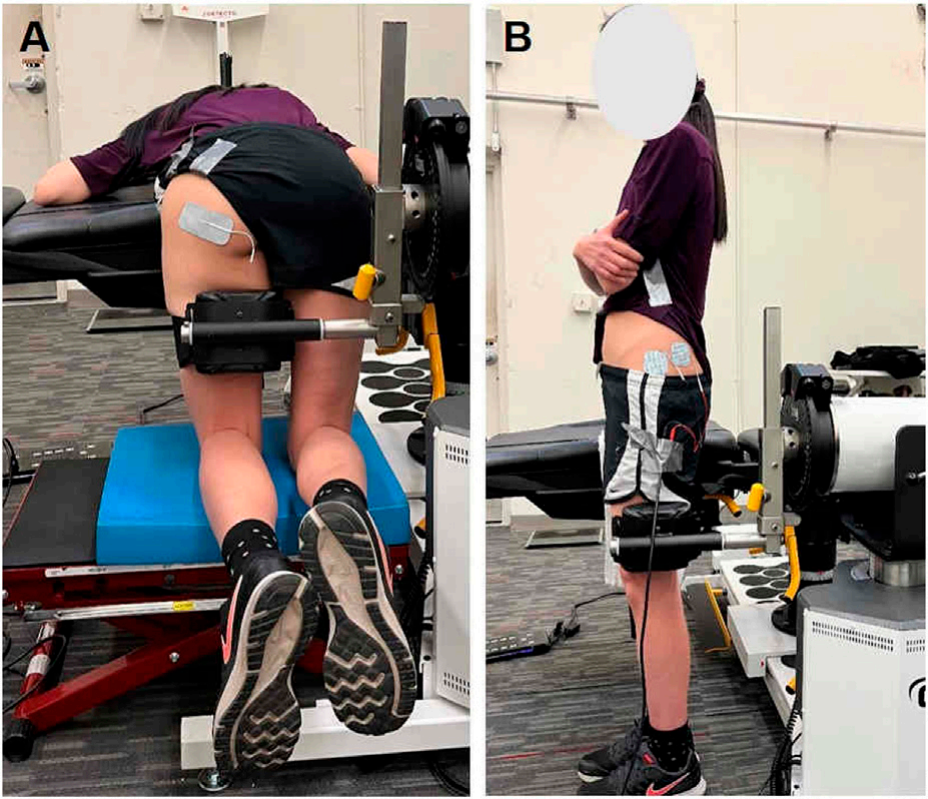
Participant positioning and electrode configurations for central activation ratio (CAR) assessments of the **(A)** gluteus maximus and **(B)** gluteus medius.

We assessed the CAR of the gluteal muscles using the SIB technique (Glaviano et al., 2019; Glaviano and Norte, 2022). Specifically, the gluteus maximus was examined while participants lie prone with 90 degrees of hip flexion and 90 degrees of knee flexion, and performed a MVIC by extending the hip against the dynamometer attachment arm. To assess CAR in the gluteus maximus, two adhesive electrodes were placed just inferior to the posterior gluteal line of the ilium and medial to the greater trochanter along the line of its insertion to the iliotibial band (Figure 1A). For assessment of CAR in the gluteus medius, participants stood on their non-test limb, and performed MVIC by abducting their test hip into the dynamometer attachment arm. The electrodes for the gluteus medius were placed at the area superior to the greater trochanter and the central area of the most superior aspect of the muscle (Figure 1B) (Te et al., 2017; Glaviano et al., 2019).

Prior to recording, a practice MVIC trial was performed, followed by a 2-minute rest period. The torque generated by the muscles and electrical stimulation was recorded by a motored dynamometer (Humac Norm, Computer Sports Medicine Inc., Stoughton, MA, United States). Participants were instructed to gradually increase their torque level to reach their MVIC within a span of 2 seconds. Once the torque reached a plateau during MVIC, a manually triggered brief electrical stimulus was delivered to the target muscle using a biphasic constant current stimulator (DS8R, Digitimer^®^, Welwyn Garden City, United Kingdom) and train generator (DS2A, Digitimer^®^, Welwyn Garden City, United Kingdom). The electrical stimulus consisted of a 100 ms train of 10 square-wave pulses of 600 us pulse width at 100 Hz, aiming to increase the torque output by recruiting the un-recruited motor units (Norte et al., 2015; Gilfeather et al., 2019; Glaviano and Norte, 2022). Manually triggering of electrical stimulation was chosen to reduce the number of MVIC trials required for CAR assessment, and to reduce possible fatigue of the participants. All participants received verbal encouragement during MVIC. To minimize fatigue, each participant performed at least two trials but no more than five MVIC trials, with a 2-minute rest period between trials for each muscle group. Additional rest was provided upon request.

### 2.3 Data analysis

#### 2.3.1 Calculation of gluteal central activation

**FIGURE 2:**
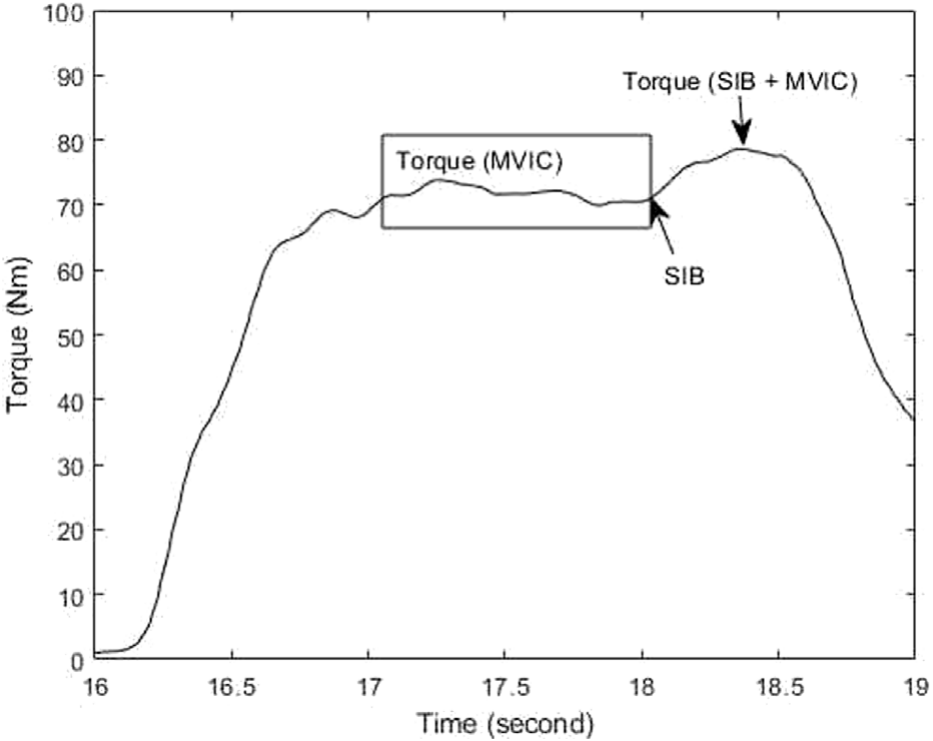
Central activation ratio (CAR) calculation during the superimposed burst (SIB) technique.

The central activation profile of hip muscles was analyzed using a custom MATLAB code (MathWorks. MATLAB. Natick, MA, United States), which involved the calculation of the CAR using the equation below (Glaviano et al., 2019; Glaviano and Norte, 2022). The torque during MVIC was defined as the torque output during the 100-millisecond window prior to the electrical stimulation, while the torque during SIB and MVIC was defined as the highest torque generated after the delivery of the electrical stimulation (as shown in Figure 2) (Glaviano et al., 2019; Glaviano and Norte, 2022). The CAR values for the gluteus medius and gluteus maximus from the collected trials were averaged and used for statistical analyses.

$$CAR=\frac{Torque\;\left(MVIC\right)}{Torque\;\left(SIB+MVIC\right)}\times 100\%$$

#### 2.3.2 Calculations of frontal plane kinematics during weight-bearing activities

The videos of participants were analyzed using Kinovea software (version 0.8.15, Kinovea, Bordeaux, France). Four primary measures were extracted from the video footage: trunk lean angle (TLA), knee frontal plane projection angle (FPPA), hip FPPA, and dynamic valgus index (DVI). These angles were measured at the peak of knee flexion (Scholtes and Salsich, 2017; Ho et al., 2024). The measurement process began by drawing a vertical reference line extending upward from the ipsilateral ASIS. To define the pelvic segment, a line was drawn connecting the markers on the bilateral ASIS landmarks. The thigh segment was represented by a line from the midpoint of the patella to the ipsilateral ASIS, while the shank segment was defined by a line from the midpoint of the patella to the midpoint of the ankle (Ho et al., 2024).

**FIGURE 3:**
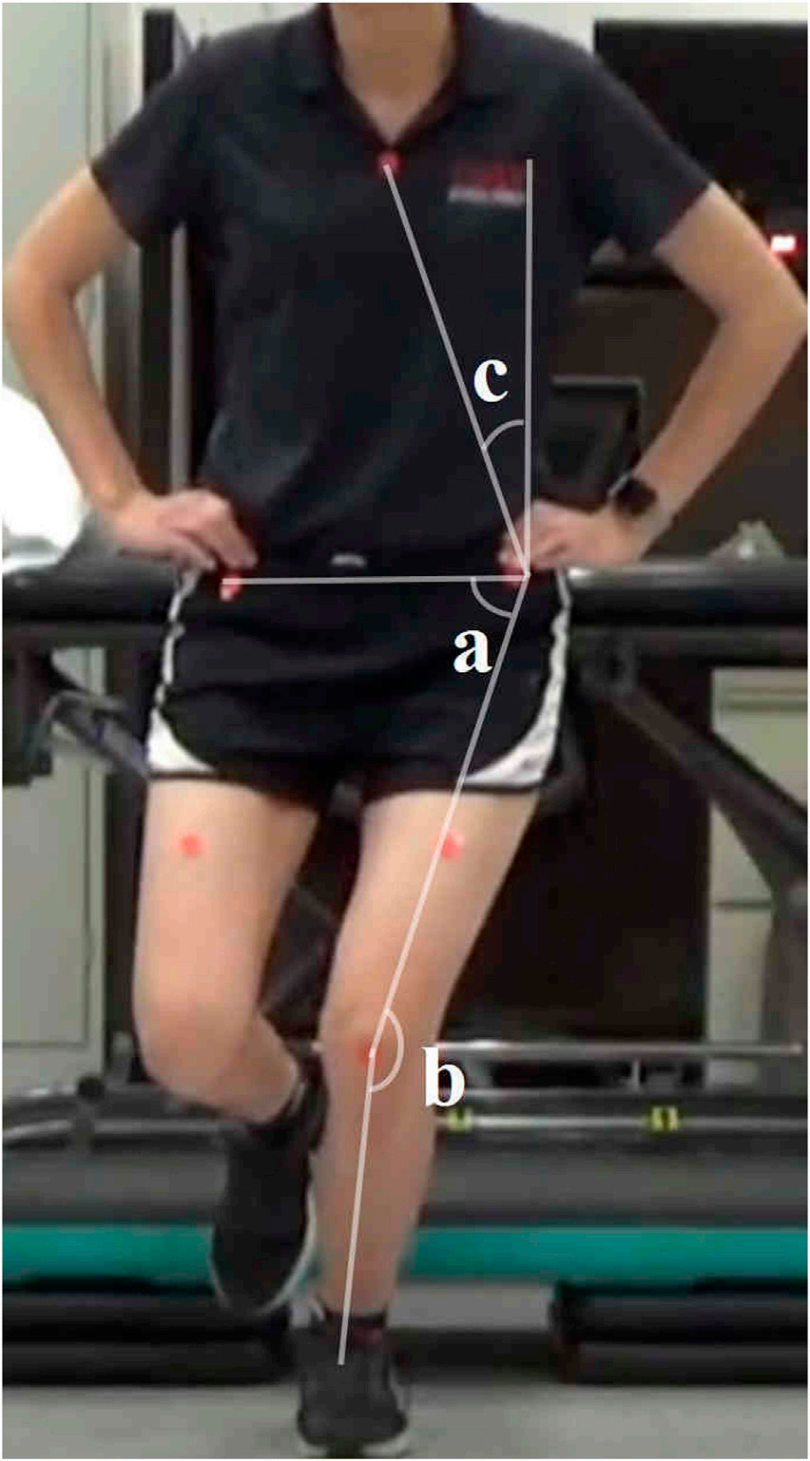
Two-dimensional frontal plane kinematics measured during single leg squat. TLA = c; hip FPPA = 90 degrees - a; knee FPPA = 180 degrees - b; DVI = a + b. Abbreviations: FPPA = frontal plane projection angle; DVI = dynamic valgus index; TLA = trunk lean angle.

The TLA was calculated as the angle between the vertical reference line and the line connecting the ipsilateral ASIS to the sternal marker (Dingenen et al., 2014). A lower TLA indicates a greater lean of the trunk towards the testing limb. The knee FPPA was determined by subtracting the angle between the thigh and shank segments from 180°. A higher knee FPPA reflects greater knee valgus in the testing limb (Scholtes and Salsich, 2017). The hip FPPA was calculated by subtracting the angle between the pelvic and thigh segments from 90°. A higher hip FPPA signifies increased hip adduction of the testing limb (Scholtes and Salsich, 2017). The DVI, which accounts for both knee valgus and hip adduction, was computed as the sum of knee FPPA and hip FPPA (Scholtes and Salsich, 2017) (Figure 3). A higher DVI indicates a greater overall degree of knee valgus and/or hip adduction. For each task, these four angles were measured from three repetitions, and the average value for each task was used in the statistical analysis.

### 2.4 Statistical analysis

The primary outcome measures included the CAR of the gluteus maximus and gluteus medius, as well as the frontal plane kinematics during five weight-bearing tasks: single leg squat, single leg hop, single leg landing, forward step-down, and lateral step-down. Independent t-tests were conducted to compare CAR measurements of the gluteus maximus and gluteus medius between the control and PFP groups. Pearson correlation coefficients were used to assess the relationships between the CAR of the gluteus maximus and gluteus medius and the frontal plane kinematics of the trunk and lower extremities (including knee FPPA, hip FPPA, DVI, and TLA) across all participants during the five weight-bearing tasks. Additionally, the correlations between gluteal CARs and the AKPS were examined for individuals with PFP using Pearson correlation coefficients. Correlations were classified as follows: small (0.1–0.3), moderate (0.3–0.5), large (0.5–0.7), very large (0.7–0.9), and extremely large (greater than 0.9) (Hopkins et al., 2009). All statistical analyses were conducted using the Statistical Package for the Social Sciences (version 23.0; IBM Corporation, Armonk, NY). A significance level was established with a threshold of p ≤ 0.05.

## 3 Results

### 3.1 Participant characteristics

The procedure was successfully completed with 10 participants experiencing PFP and 12 pain-free controls. Independent t-tests indicated that there were no differences between groups in age, body mass index (BMI) and activity level. The chi-square test confirmed that the sex distribution was similar between the two groups (Table 1).

**TABLE 1:** Participant characteristics of the patellofemoral pain (PFP) and control groups.

	PFP	Controls	P Value
Age (years)	22.6 ± 2.8	24.2 ± 1.8	0.052
Sex	4 males; 6 females	4 males; 8 females	0.746
Body Mass Index (BMI) (kg/m^2^)	23.5 ± 3.1	24.0 ± 4.2	0.419
Physical activity level (MET. min/week)	4,205 ± 2,575	2,510 ± 1,732	0.160
Anterior Knee Pain Scale	81.4 ± 9.3	N/A	N/A
Duration of symptoms (months)	35.4 ± 27.2	N/A	N/A
Average pain	2.9 ± 1.7	N/A	N/A

Data is presented as mean ± standard deviation.

### 3.2 Gluteal central activation

**FIGURE 4:**
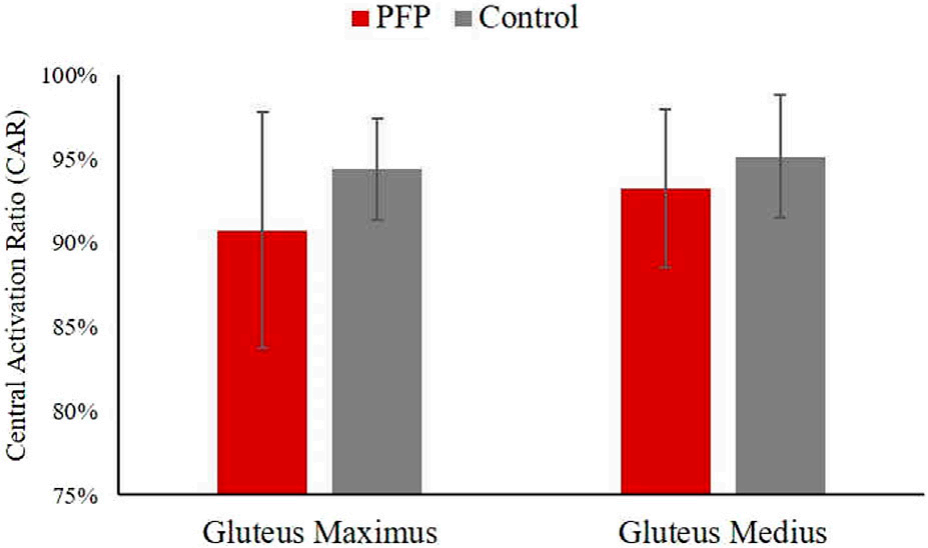
Comparisons of central activation ratio of the gluteus maximus and gluteus medius. Data is presented as mean ± standard deviation. PFP, patellofemoral pain.

Independent t-tests revealed no statistically significant difference in the CAR of the gluteus maximus between the PFP group (90.8% ± 7.0%) and the control group (94.4% ± 7.0%) (p = 0.067), despite an observed 3.6% decrease in CAR in the PFP group. This lack of statistical significance is likely attributable to the large standard deviation observed in the gluteus maximus CAR within the PFP group, suggesting substantial variability in central activation levels among individuals. Similarly, there was no statistically significant difference in the CAR of the gluteus medius between the PFP group (93.3% ± 4.7%) and the control group (95.2% ± 3.7%) (p = 0.151) (Figure 4). Therefore, our hypothesis that individuals with PFP would exhibit lower CAR values in both the gluteus maximus and gluteus medius compared to controls was not fully supported. While individuals with PFP tended to have lower CAR in the gluteus maximus, it did not reach statistical significance.

### 3.3 Correlations between gluteal central activation and kinematics/functional outcomes

Pearson correlation coefficient analysis revealed a statistically significant large correlation between the CAR of the gluteus maximus and the TLA during single leg landing across all participants (R = 0.533, p = 0.006; Table 2). No significant associations were observed between gluteal CAR values and the kinematics of other tasks. Additionally, there was a significant correlation between the CAR of the gluteus maximus and scores on the AKPS in individuals with PFP (R = 0.790, p = 0.003; Table 2). A significant correlation was also found between the CAR of the gluteus medius and AKPS scores (R = 0.584, p = 0.038; Table 2). Consequently, our hypothesis that lower gluteal CAR would correlate with altered FPPA of the trunk and lower extremities, as well as reduced function, was partially supported. Specifically, we observed a relationship between gluteus maximus CAR and TLA during single leg landing, as well as between gluteal CAR and AKPS scores.

**TABLE 2:** Correlations between central activation ratio (CAR) of gluteal muscles, frontal plane kinematics and function.

	CAR of gluteus maximus		CAR of gluteus medius	
	R	P value	R	P value
Single Leg Squat				
Trunk Lean Angle	0.042	0.428	0.283	0.101
Hip FPPA	−0.201	0.191	0.206	0.179
Knee FPPA	−0.214	0.176	−0.164	0.233
Dynamic Valgus Index	−0.223	0.166	−0.010	0.482
Single Leg Hopping				
Trunk Lean Angle	0.228	0.160	0.007	0.487
Hip FPPA	−0.122	0.299	0.003	0.495
Knee FPPA	0.006	0.490	0.017	0.471
Dynamic Valgus Index	−0.066	0.389	0.010	0.482
Single Leg Landing				
Trunk Lean Angle	0.533	0.006*	0.305	0.083
Hip FPPA	−0.018	0.469	0.039	0.432
Knee FPPA	0.082	0.362	0.150	0.253
Dynamic Valgus Index	0.040	0.432	0.109	0.314
Forward Step Down				
Trunk Lean Angle	0.252	0.135	0.194	0.194
Hip FPPA	−0.04	0.494	0.022	0.461
Knee FPPA	0.40	0.432	−0.008	0.486
Dynamic Valgus Index	0.023	0.461	0.006	0.490
Lateral Step Down				
Trunk Lean Angle	0.080	0.365	0.248	0.132
Hip FPPA	−0.167	0.235	0.138	0.270
Knee FPPA	−0.003	0.495	0.120	0.298
Dynamic Valgus Index	−0.087	0.353	0.143	0.262
Anterior Knee Pain Scale	0.790	0.003*	0.584	0.038*

Abbreviations: CAR, central activation ratio; FPPA, frontal plane projection angle.

* Indicates a statistically significant difference using a Pearson correlation coefficient analysis.

## 4 Discussion

Our primary aim was to compare the CAR of the gluteus medius and gluteus maximus between individuals with and without PFP. A secondary objective was to examine the relationship between CAR of the gluteal muscles and the frontal plane kinematics of the trunk and lower extremities during five weight-bearing activities and patellofemoral joint function. Our hypotheses were partially supported. We observed a 3.6% decrease in gluteus maximus CAR in individuals with PFP compared to controls, but the difference did not reach statistical significance. Gluteus medius CAR was not different between groups. With respect to our secondary aim, higher CAR values for gluteus maximus and gluteus medius were associated with higher AKPS scores. Additionally, reduced gluteus maximus CAR was linked to lower TLA, reflecting greater ipsilateral trunk lean toward the standing limb during movement. This may represent a compensatory strategy to decrease external hip moments and reduce the demand on hip muscles during single leg landing.

The current literature demonstrates considerable variability in CAR values among individuals with and without PFP. In asymptomatic individuals, Gilfeather et al. (2019) reported CAR values of 96.4% for the gluteus medius and 86.9% for the gluteus maximus, while an unpublished study (Samuel, 2022) reported an even higher gluteus medius CAR of 98.4% in asymptomatic individuals. In comparison, in our study, our observed gluteus medius CAR of 95.15% in pain-free controls aligned with previous research, whereas our observed gluteus maximus CAR of 94.40% was higher than previously reported values in pain-free groups. Among those with PFP, Glaviano and Norte (Glaviano and Norte, 2022) found CAR values of 90.5% for the gluteus medius and 84.0% for the gluteus maximus in females with PFP, whereas Samuel (Samuel, 2022) reported a higher gluteus medius CAR of 95.9% in females with PFP. Our study’s CAR values for the PFP group (gluteus maximus = 90.8 ± 7.0%; gluteus medius = 93.3 ± 4.7%) fell within these reported ranges. Importantly, consistent with prior research (Glaviano and Norte, 2022), we also observed higher CAR for gluteus medius compared to gluteus maximus in persons with PFP. This disparity in hip muscle activation may contribute to increased knee valgus in persons with PFP, as insufficient strength or neuromuscular control in the hip abductors could impair their ability to abduct the thigh during weight-bearing activities, leading to altered movement patterns.

In comparing gluteal CAR between individuals with and without PFP, unpublished data by Samuel (Samuel, 2022) reported a 2.5% reduction in the CAR of the gluteus medius among those with PFP (PFP = 95.9%; control = 98.4%). Our study expanded on this by including an analysis of the gluteus maximus. Although the difference in CAR for the gluteus maximus and gluteus medius between individuals with and without PFP did not reach a statistical significance in our study, comparisons for the gluteus maximus approached significance, indicating a possible lower gluteus maximus CAR in participants with PFP compared to pain-free controls (p = 0.067). Despite reaching our targeted sample size based on power estimates, we did not observe a statistically significant difference between groups. A key factor contributing to this outcome was likely the wide variability in gluteal CAR values among participants, with gluteus maximus CAR ranging from 80.5% to 99.7%, and gluteus medius CAR from 84.4% to 98.3%. These observations suggest the potential presence of subgroups within the PFP population, where some individuals may not exhibit central activation deficits despite having PFP. Therefore, identifying these subgroups within the PFP population is crucial. Since not all individuals with PFP may present with diminished gluteal CAR, further research is needed to identify subgroups of PFP patients with central activation deficits, explore how these deficits relate to movement impairments, and establish a threshold for defining reduced gluteal CAR.

A key finding from our study was the correlation between lower CAR values and increased ipsilateral trunk lean during the single leg landing task. This suggests that participants may lean toward the stance limb to reduce external hip moments, possibly as a compensatory strategy to offload the demand on the hip muscles. Glaviano and Norte (Glaviano and Norte, 2022), using three-dimensional (3D) motion analysis, reported an association between lower gluteus maximus CAR values and greater hip adduction during single leg squat. However, their study did not examine trunk lean, focusing solely on hip kinematics during the squat. The difference in tasks and methodological approaches may explain the divergence in findings between our study and theirs. Specifically, the 3D motion analysis employed by Glaviano and Norte offers enhanced precision in capturing complex movement patterns, potentially explaining why they observed relationships with hip adduction that were not evident in our data. When comparing our observations with Samuel’s unpublished study, which utilized a similar 2D motion analysis approach for the lower extremities during a forward step-down task, neither study identified a significant relationship between 2D frontal plane kinematics of the lower extremities and gluteal CAR values. These discrepancies across studies highlight the complexity of the relationships between gluteal CAR and movement kinematics. This suggests the need for future research utilizing 3D motion analysis to investigate how gluteal activation patterns influence kinematics of both the lower extremity and trunk in persons with PFP. Examining a wider range of weight-bearing tasks, including those that challenge frontal and transverse plane stability, could offer deeper insights into compensatory movement strategies and their implications for individuals with PFP. Additionally, future studies should aim to explore the potential influence of individual variability, such as differences in pain severity, activity levels, or neuromuscular adaptations, which may contribute to the observed variability in gluteal CAR and its relationship with kinematics.

Our study found that lower CAR in both the gluteus maximus and gluteus medius were linked to decreased function, as measured by the AKPS. These results highlight the crucial role of central activation in the gluteal muscles for maintaining proper patellofemoral joint function in persons with PFP. This is consistent with the findings of Glaviano and Norte (Glaviano and Norte, 2022), who identified a correlation between gluteus medius CAR values and the Fear-Avoidance Belief Questionnaire-Physical Activity scores. Their research suggests that individuals with PFP who have lower gluteus medius CAR values are more likely to experience greater fear avoidance toward physical activity. These insights highlight the potential role of gluteal central activation in managing both functional limitations and psychological fear associated with PFP.

A potential intervention to address decreased central activation of the gluteal muscles in persons with PFP is transcranial direct current stimulation (tDCS), a non-invasive brain stimulation technique that delivers a direct weak electric current to the brain, and can modulate cortical excitability (Nitsche and Paulus, 2000; Lefaucheur et al., 2017), improve motor function (Hummel et al., 2005; Liang et al., 2020), or alleviate pain (Yang et al., 2024). The modulatory effect is influenced by the positioning and polarity of the scalp electrodes. Anodal stimulation enhances cortical excitability, while cathodal stimulation reduces it, and bimodal stimulation simultaneously increases excitability in the region beneath the anode and decreases excitability in the region beneath the cathode (Nitsche and Paulus, 2000). Our recent research study has demonstrated the feasibility of using bimodal tDCS to target gluteal corticomotor function in combination with hip muscle strengthening in individuals with PFP (Ho et al., 2024). In our earlier study, we used the bimodal montage, where tDCS was applied with the anode positioned over the primary motor cortex contralateral to the more painful limb and the cathode over the ipsilateral motor cortex, aiming to enhance motor cortex function and optimize its effects. Future research should explore the use of tDCS in individuals with PFP who exhibit reduced gluteal CAR, examining its potential clinical benefits in improving gluteal muscle activation, hip muscle performance, trunk and lower extremity kinematics, and overall functional outcomes.

This study has a few limitations. First, the findings have limited generalizability to the broader population due to the relatively young age of the participants, with a mean age of 22.6 years in the PFP group and 24.2 years in the control group. As such, the results may not fully reflect the variations seen in a broader PFP population that includes individuals of different ages, and caution should be exercised when attempting to apply these findings to individuals outside the specific age range studied. Further research including a wider age range would be necessary to better understand the relationship between gluteal CAR and PFP across different age groups. Additionally, participants in the PFP group exhibited a wide range of physical activity levels, with most being highly active, as indicated by their GPAQ scores. However, no significant difference in overall physical activity was observed between the PFP group and the control group. It is also interesting to note that GPAQ scores were not correlated with gluteal muscle CARs in this study. This may be due to the fact that, while the GPAQ assesses various aspects of physical activity in daily life, it does not specifically target activities that involve hip muscle training.

## 5 Conclusion

Our study did not reveal significant group differences in gluteal CAR, although individuals with PFP showed a tendency for lower gluteus maximus CAR. In addition, greater gluteal central activation was associated with better function in individuals with PFP. The observed relationship between lower gluteus maximus CAR and ipsilateral trunk lean during single leg landing suggest a compensatory strategy aimed at reducing external hip moments and offloading the demand on hip muscles. This highlights the potential role of neuromuscular adaptations in the movement patterns of individuals with PFP.

Future research should prioritize large-scale studies to better understand the heterogeneity of gluteal central activation within the PFP population. Specifically, efforts should focus on identifying subgroups characterized by diminished gluteal central activation and exploring the clinical implications of such deficits. Additionally, studies should investigate the biomechanical and neuromuscular mechanisms linking gluteal central activation to movement outcomes, with a specific focus on weight-bearing tasks that require dynamic stability. Furthermore, longitudinal research could examine the effects of targeted interventions, such as neuromuscular training, cortical priming using tDCS, or hip strengthening programs, on gluteal central activation and movement biomechanics. Investigating whether these interventions can mitigate abnormal kinematics or improve functional outcomes would provide valuable insights for optimizing rehabilitation strategies for individuals with PFP.

## Data Availability

The raw data supporting the conclusions of this article will be made available by the authors, without undue reservation.
